# Clinical Profile of Pediatric Solid Tumors: Experience From a Tertiary Care Center in Ethiopia

**DOI:** 10.1002/cnr2.70222

**Published:** 2025-05-08

**Authors:** Mamude Dinkiye, Deme Abdissa, Tadele Hailu, Aziza T. Shad, Yoram Unguru

**Affiliations:** ^1^ Department of Pediatrics and Child Health School of Medicine, Saint Paul's Hospital Millennium Medical College Addis Ababa Ethiopia; ^2^ Division of Pediatric Hematology/Oncology The Herman and Walter Samuelson Children's Hospital at Sinai Baltimore Maryland USA; ^3^ The Aslan Project Washington, DC USA; ^4^ Johns Hopkins Berman Institute of Bioethics Baltimore Maryland USA

**Keywords:** clinical profile, Ethiopia, pediatric solid tumors

## Abstract

**Background:**

Pediatric solid tumors are a significant health challenge worldwide, especially in low‐ and middle‐income countries such as Ethiopia, where healthcare infrastructure is limited and treatment modalities are scarce.

**Aims:**

This study aims to understand the epidemiological characteristics of these tumors and short‐term treatment outcomes.

**Methods and Results:**

A retrospective study spanning a period of 2 years and 8 months was conducted among all children below age 15 years admitted to St. Paul's Hospital Millennium Medical College hemato‐oncology unit with a pediatric solid tumor. Data from patient charts was extrapolated and analyzed using SPSS version 29. A total of 173 pediatric solid tumor patients were identified over the study period. 22.6% of patients were treated in the first year, 34.6% of patients were treated in the second year, and 42.8% of patients were treated in the last 8 months of the study period. 56.1% of them were males. Most patients came from the Oromia region. The most frequent solid tumors were retinoblastoma, Wilms tumor, and rhabdomyosarcoma. Eighty‐six patients are still on treatment, 23 patients achieved complete remission, 4 relapsed, 2 were defaulters, 5 patients were lost to follow‐up, 21 died, 5 were referred to other hospitals, and 10 opted against treatment.

**Conclusion:**

The study reveals a rising trend in childhood solid tumor cases over the years. A significant proportion of patients achieved remission, whereas most remain under treatment or follow‐up care. A relatively small percentage experienced relapses, with some cases of defaulters, loss to follow‐up, and a few instances of mortality. Implementing early detection strategies and community‐based awareness programs could improve outcomes by encouraging timely diagnosis and intervention.

**Abbreviations**
ED: emergency department
HIC: high‐income countries
LMIC: low‐ and middle‐income countries
OPD: outpatient department
PI: principal investigator
SPHMMC: Saint Paul's Hospital Millennium Medical College
SPSS: Statistical Package for Social Sciences
WHO: World Health Organization

## 1 Introduction

Childhood cancer remains the leading cause of disease‐related mortality in children. Solid tumors account for approximately 30% of childhood cancers [1]. Although childhood cancer remains a rare disease, since 1975, overall incidence has gradually increased [2]. Worldwide, the annual number of new childhood cancers exceeds 200 000, with more than 80% occurring in low‐ and middle‐income countries (LMIC) [2]. In a recent summary analyzing cooperative group studies from high‐income countries (HICs), 5‐year event‐free survival and overall survival rates for localized tumors have improved to approximately 73% and 84%, respectively, with 5‐year survival rates for certain cancers such as Hodgkin lymphoma (HL) and retinoblastoma approaching 95% [3, 4]. Between 1975 and 2010, childhood cancer survival increased by more than 50% [5]. This success can be attributed to several factors. These include enrollment of patients into well‐designed prospective clinical trials, systematic collection of tissue to better define the biology of the disease, availability of more effective chemotherapy agents, use of multimodal therapy, better supportive care, and more refined diagnostic imaging methods that accurately define the extent of disease [6].

Recent studies have shown that this success in survival can be replicated in the developing world through shared expertise and innovative strategies [4]. Compared to infectious diseases, which have been and remain a major public health concern in developing nations, malignancies have taken a backseat. Overcoming this mindset is one challenge to improving outcomes for children with cancer in LMIC [7].

St. Paul Hospital Millennium Medical College (SPHMMC) is one of the few tertiary healthcare institutions in the capital city of Ethiopia, Addis Ababa, which offers comprehensive care to pediatric cancer patients. Despite the existence of standard management protocols, limited information is available on the clinical profile, treatment patterns, and short‐term outcomes of pediatric patients with solid tumors in this tertiary hospital. Accurate data will better enable decision‐makers to evaluate program requirements, prioritize actions, and track advancements effectively. Moreover, although some studies have reported the incidence and histopathological classification of solid tumors in Ethiopian children, these studies have not provided insights into the clinical profile and treatment outcomes of these tumors. Therefore, this research aims to evaluate the clinical profile and short‐term treatment outcomes of pediatric patients with solid tumors at SPHMMC.

## 2 Methods

### 2.1 Study Design, Area, and Period

A Hospital‐based retrospective cross‐sectional study of pediatric patients with solid tumors treated at SPHMMC between March 2021 and December 2023. Ethiopia is in East Africa and designated as a LIC by the World Bank with limited advanced health care facilities and expertise. SPHMMC is one of the few government hospitals in the capital Addis Ababa, which serves as a general/referral center for more than 5 million people. The hospital was established in 1969 by the late emperor Haile Selassie in collaboration with the German Evangelical Church and is currently governed by a board under the federal ministry of health. In addition to an inpatient capacity of more than 700 beds, on average, 2000 emergency and outpatient clients are seen daily in the hospital. Pediatric oncology patients are seen at the emergency department (ED), oncology follow‐up Clinic, and oncology ward, which was established in March 2021. Initially a 6‐bed oncology ward for patients with solid tumors only, it has now expanded to an 18‐bed ward caring for both solid tumors and hematologic malignancies. Currently, SPHMMC has two pediatric hematologist‐oncologists, pediatric residents, and trained oncology nurses.

### 2.2 Study Population and Sampling Procedures

The case files of all children age 15 years and younger with solid tumors who were admitted to the pediatric ward between March 2021 and December 2023 were reviewed.

### 2.3 Data Collection

Data extracted from the case files/charts included sociodemographic characteristics, diagnosis, disease stage, disease site, risk stratification, and treatment modality. Diagnosis was confirmed based on evaluation by a pediatric hematologist‐oncologist supplemented with histopathological results (pathological biopsy, immunohistochemistry) and relevant imaging modalities (CT/MRI/PET‐CT scan).

Following treatment completion, patients were followed in the outpatient pediatric hematology/oncology department to assess remission status and treatment outcomes. Remission refers to a state where there is a significant reduction or disappearance of cancer by using various criteria such as clinical response (absence or reduction of cancer‐related symptoms), radiological (no visible tumor or reduced size on CT or MRI) and laboratory findings (normal tumor markers, no detectable cancer cells in the blood or bone marrow). Relapse refers to the return of cancer after a period of improvement or remission. A “defaulter” refers to a patient who discontinues or fails to complete prescribed cancer‐directed treatment or follow‐up care. For patients who could not attend follow‐up in person, telephone follow‐up was conducted to determine their short‐term outcomes.

### 2.4 Data Analysis

After verifying for completeness and consistency, data was coded and entered into Epi Info version 7 and exported to SPSS version 29.0 for analysis. Descriptive statistics were used to analyze and present patient data.

### 2.5 Ethical Considerations

Ethical clearance was obtained from the SPHMMC Ethical Review Committee. Written consent was obtained from the hospital administration and the Department of Pediatrics and Child Health. Confidentiality was ensured by using codes instead of patient names during data registration.

## 3 Results

### 3.1 Demographic Data

**FIGURE 1:**
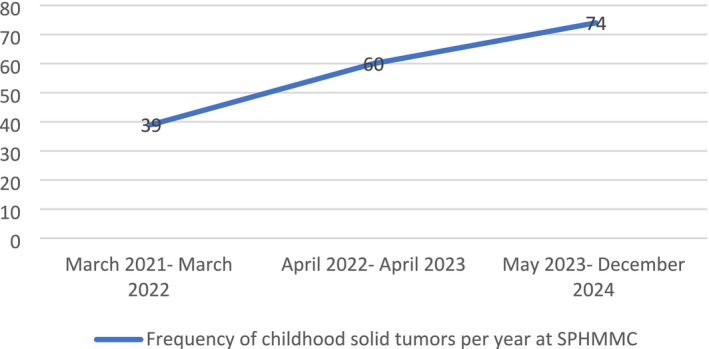
Frequency of childhood solid tumors per year at SPHMMC.

From March 2021 to December 2023, a total of 173 pediatric solid tumor patients were registered at SPHMMC. Of these, 54.8% were male and 45.2% were female (M:F ratio of 1.21:1). Most patients (55.5%) were from the Oromia region, followed by Addis Ababa (18.5%) and the Amhara region (11.6%). Six percent were infants (< 1 year), 52% were 1–5 years old, 30% were 5–10 years old, and 12% were 10–15 years old (Table 1). Thirty‐nine patients were seen during the first year of the study (22.6%), 60 in the second year (34.6%), and 74 patients were seen in the last 8 months of the study period (42.8%) (Figure 1).

**TABLE 1:** Pediatric solid tumor distribution at SPHMMC.

Tumor	Sex		Age distribution (in years)			
	M	F	< 1	1–5	5–10	> 10
Retinoblastoma	33	20	11	40	2	0
Wilms tumor	14	16	2	18	7	3
Rhabdomyosarcoma	12	11	0	10	12	1
Hodgkin lymphoma	15	3	0	2	8	8
Germ cell tumor	3	9	0	3	5	4
Non‐Hodgkin lymphoma	1	5	0	1	2	3
Ewing sarcoma	3	2	0	1	3	1
Nasopharyngeal carcinoma	2	3	0	1	1	3
Neuroblastoma	1	2	0	1	2	0
Hepatoblastoma	2	0	1	1	0	0

### 3.2 Tumor Distribution

**FIGURE 2:**
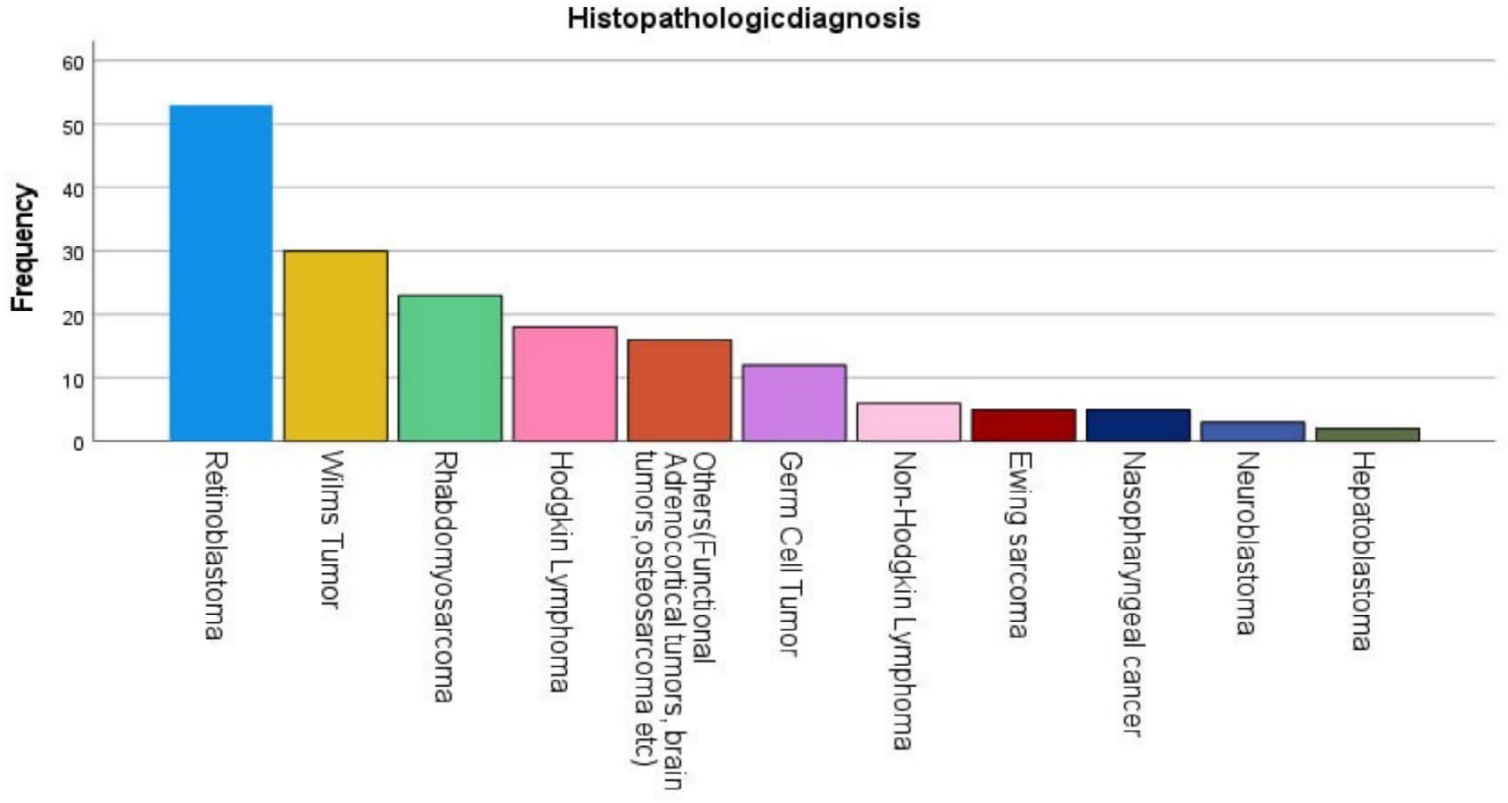
Major childhood solid tumor distribution at SPHMMC.

In descending order of frequency, the most frequent solid tumors were retinoblastoma (30.6%) followed by Wilms tumor (17.3%), rhabdomyosarcoma (13.3%), HL (10.4%), other tumors (functional adrenocortical tumors, brain tumors, osteosarcoma) (9.2%), germ cell tumors (6.4%), non‐Hodgkin lymphoma (4%), nasopharyngeal cancer (2.9%), neuroblastoma (1.7%) and hepatoblastoma (1.2%) (Figure 2).

### 3.3 Individual Tumor Characteristics

*Retinoblastoma* (*n* = 53) occurred more frequently in males (62%) and mostly presented in children in the 1–5‐year age group (75%) followed by infants up to 12 months (21%) and children ages 5–10 years (4%); no retinoblastoma was seen in children 10 years and older. The most common presenting feature was leukocoria (59%) followed by buphthalmos (23.1%), conjunctival injection (13.5%) and strabismus (3.8%). Nearly half of patients presented for care within 6 months of onset of symptoms (47.2%), almost one‐quarter (22.6%) presented within 6–12 months, 18.9% did so less than 1 month after symptoms began, and 11.3% did so > 12 months. Right‐eye disease occurred in 43.4%, 37.7% involved the left eye, and 18.9% were bilateral. Most tumors were diagnosed in Reese–Ellsworth Group E (70.2%), followed by Group C (14.9%), Group D (12.8%), and Group B (2.1%). Over two‐thirds of patients (69.2%) were treated with chemotherapy and enucleation; chemotherapy alone (23.1%), chemotherapy with laser therapy (5.8%) and chemotherapy with radiotherapy (1.9%). At the time of data collection, 30 patients were on treatment (56.6%), 13 had achieved remission (24.5%), 2 were lost from follow‐up (3.8%), 1 was referred to another hospital (1.9%), 1 left against medical advice (1.9%) and 6 patients died (11.3%).

*Wilms tumor* (*n* = 30) was more common in females (53%) and mostly presented in children in the 1–5‐year age group (60%) followed by ages 5–10 years (23%), above 10 years (11%) and infants up to 12 months (6%). An abdominal mass was the most common presenting feature seen in 96.7% of patients followed by abdominal pain (3.3%). Most patients presented for care within 6 months of the onset of symptoms (70%). Twenty percent did so within < 1 month and 10% did so within 6–12 months of symptom onset. More than half of tumors (53.3%) occurred on the left side, 30% on the right side, and 16.7% were bilateral. Half of tumors were Stage IV at diagnosis (50%), followed by Stage V (16.7%), Stage I and Stage III (13.3% each), and Stage II (6.7%). Twenty‐one patients were treated with chemotherapy alone (75%), 6 patients with chemotherapy and surgery (21.4%), and 1 patient with surgery alone (3.6%). Thirteen patients were on treatment at the time of data collection (43.3%), 2 patients relapsed (6.7%), 7 left against medical advice (23.3), 1 achieved remission (3.3%), 1 patient was referred to another hospital (3.3%) and 6 died (20%).

*Rhabdomyosarcoma* (*n* = 23) occurred nearly equally among genders: Males (52%) and mostly presented in children in the 5–10‐year age group (52%), followed by children ages 1–5 years (44%) and those above 10 years (4%) with no cases in infants less than 12 months of age. The most frequently occurring site was the head and neck (60.9%), followed by the genitourinary (17.4%), extremities (8.7%), gastrointestinal (8.7%), and other sites (4.3%). Most patients presented for care within 6–12 months of the onset of symptoms (56.5%); 4.6% did so within 1–6 months, and 8.7% had symptoms for > 12 months. Most tumors (10 patients) were diagnosed in Stage III (50%), followed by 5 children with Stage IV disease (25%), 4 with Stage II (20%) and 1 with Stage I (5%). More than half were diagnosed with high risk (52.2%), followed by intermediate risk (39.1%) and low risk (8.7%). Most patients were treated with chemotherapy alone (61.9%). Chemotherapy and surgery were pursued in 38.1% of patients. Twelve patients were on treatment at the time of data collection (52.2%). One patient completed treatment and achieved remission (4.3%); 2 patients defaulted treatment for more than 1 month (8.7%); 2 patients were referred to other hospitals (8.7%); 1 patient relapsed (4.3%); and 5 died (21.7%).

*Hodgkin lymphoma* (*n* = 18) was most common in males (83%) and presented equally in children in the 5–10‐year and above 10‐year age groups (44%) each. HL occurred less frequently in younger age groups, 12% in children 1–5 years and none in the infancy age group. The commonest presenting feature is lymphadenopathy (76.5%) followed by lymphadenopathy, mediastinal mass, B symptoms combined (11.8%), mediastinal mass alone (5.9%) and B symptoms alone (5.9%). Over half of patients presented for care within 6–12 months of onset of symptoms (52.9%); nearly a third did so > 12 months (29.4%), with 11.8% seeking care within 1–6 months and 5.9% in less than 1 month. Predominant histology was classical HL (82.4%) followed by nodular lymphocyte predominant HL (17.6%). Stage III disease was most frequent (62.5%), followed by Stage IV (18.8%), Stage II (12.5%), and Stage I (6.3%). Most patients were stratified as intermediate risk (52.9%), with a significant percentage considered as high risk (41.2%) and relatively low‐risk HL (5.9%). All patients were treated with chemotherapy. Most patients achieved remission (47.1%). At the time of data collection, nearly half were on treatment (47.1%); 5.8% were lost to follow up.

*Germ cell tumor* (*n* = 12) was more common in males (75%) and mostly presented in children aged 5–10 years (41%), followed by those above 10 years (34%) and those aged 1–5 years (25%). None were diagnosed in the infancy age group. The most common presenting feature was an abdominal mass (11 cases, 91.7%), with other symptoms including testicular enlargement and back pain (1 case, 8.3%). The duration of illness before presentation was between 1 and 6 months in 11 patients (91.7%) and 6–12 months in 1 patient (8.3%). There were 7 cases of teratoma (58.3%), 2 yolk sac tumors (16.7%), 2 ovarian tumors (16.7%), and 1 other type (8.3%). Seven patients (58.3%) were treated with both chemotherapy and surgery, 3 (25%) with chemotherapy alone, and 2 (16.7%) with surgery alone. Currently, 9 patients (75%) are still undergoing treatment, 1 (8.3%) was lost to follow‐up, 1 (8.3%) left against medical advice, and 1 (8.3%) has died.

*Non‐Hodgkin lymphoma* (*n* = 6): Most of the 6 recorded cases occurred in females (83%) and in children aged 5–10 years (83%) followed by 1–5 years (17%) with no cases among infants or children older than 10 years of age. The most common presenting feature was abdominal mass (hepatic and splenic enlargements) (83.3%) and all patients presented within 1–6 months. Burkitt lymphoma was the predominant histology (75%), followed by diffuse large B‐cell lymphoma (25%). Stage II and IV tumors occurred with equal frequency (40%) followed by Stage III (20%). Intermediate risk (66.7%) was most common, followed by high risk (33.3%) tumors. Five patients were treated with chemotherapy (83.3%) and 1 patient was treated with chemotherapy and surgery (16.7%). At the time of data collection, 4 patients were on treatment (66.7%), 1 patient was referred to another hospital (16.7%), and 1 patient died (16.7%).

*Nasopharyngeal carcinoma* (*n* = 5): Three of 5 cases occurred in males (60%) and in children in the age group of > 10 years (60%) followed by 1 case each in children ages 5–10 years and 1–5 years (20%). Neck swelling was the most common presenting feature seen in all patients (100%). Four of five patients (80%) presented within 1–6 months of onset of symptoms, and one patient did so within 6–12 months (20%). Stage IV disease was most common (80%) and Stage III was seen in 1 patient (20%). All patients were treated with chemotherapy, and 4 patients were on treatment at the time of data collection (80%), and 1 patient died (20%).

*Ewing sarcoma* (*n* = 5): Three of five cases were documented in males (60%) and mostly in children in the 5–10‐year age group (60%); 1 case each occurred in the 1–5 years and > 10 years groups (20%) each. The most common presenting feature was extremity swelling (80%). Two patients each (40%) presented within 1–6 months and 6–12 months following symptom onset, and 1 patient did so > 12 months (20%). Two patients presented with a skull lesion (40%), 2 had a spinal mass (40%), and in 1 patient, Ewing sarcoma involved the long bones (femur, tibia and humerus) (20%). Most of the patients were diagnosed in Stage IV (40%), Stage II (20%) and Stage III (20%). Chemotherapy alone was utilized in four cases (80%), whereas 1 patient was treated with chemotherapy and surgery (20%). At the time of data collection, 3 patients were on treatment (60%), 1 patient had relapsed 1 (20%), and 1 patient died (20%).

*Neuroblastoma* (*n* = 3): Two of three cases occurred in females (66%) and presented in children in the 5–10 years age group (66%) followed by one patient in the 1–5 years group (33%). Abdominal mass was present in 2 patients (66.7%) and abdominal pain in 1 patient (33.3%). Two patients (66.7%) sought care within 6–12 months of symptom onset, whereas 1 patient presented within 1 month (33.3%). Two patients were treated with chemotherapy (66.7%) and 1 patient with chemotherapy and surgery (25%). Two of the patients were on treatment (66.7%) at the time of data collection, and 1 patient left against medical advice (33.3%).

*Hepatoblastoma* (*n* = 2): Both patients were males; one was in the 1–5‐year age group and the other in the infant group. Abdominal distention was present in both patients. One patient presented within 6–12 months of onset of symptoms and the other within < 1 month. Both patients were treated with chemotherapy alone. At the time of data collection, 1 patient was on treatment, and 1 patient was lost to follow‐up.

*Other tumors* (*n* = 16): These included 2 adrenocortical tumors, 2 craniopharyngiomas, 1 glioma, 2 astrocytomas, 1 medulloblastoma, 1 osteosarcoma, 1 gastric lymphoma, 1 liver mass secondary to subcapsular hamartoma, 1 orbital lymphoma, 3 hemangiomas, and 1 lymphangioma.

### 3.4 Short‐Term Treatment Outcome

Overall, among the studied patients, 86 patients were receiving treatment at the time of data collection (55.1%), 23 had successfully entered remission (14.7%), 21 died (13.4%), 10 left against medical advice (6.56%), 5 were lost to follow‐up (3.2%), 5 were referred to another hospital (3.2%), 4 relapsed (2.56%), and 2 were defaulters (1.28%) (Table 2).

**TABLE 2:** Overall short‐term treatment outcome of pediatric solid tumors at SPHMMC.

Treatment outcome	Total	Percentage
Achieved remission/completed treatment	23	14.7%
Defaulter (> 1 month)	2	1.28%
Relapse	4	2.56%
On treatment	86	55.1%
Lost to follow‐up	5	3.2%
Death	21	13.4%
Refer to other hospitals	5	3.2%
Left against medical advice	10	6.4%

## 4 Discussion

### 4.1 Demographic Characteristics and Admission Patterns

Over the period of our study conducted at a large academic hospital in Ethiopia, the incidence of childhood solid tumors increased. This trend may be attributed to heightened public awareness, enhanced referral linkage system, and improved health‐seeking behavior within the community.

In general, childhood cancer exhibits a higher incidence among males compared with females, with the male‐to‐female ratio typically hovering around 1.2:1 in the most affluent nations [8]. The observed slight male predominance in our study is consistent with similar reports [4, 9, 10]. Nevertheless, certain tumors like retinoblastoma, Wilms tumor, osteosarcoma, and germ cell tumors demonstrate a slight preference towards females [8]. The high number of patients coming from the Oromia region seeking care at St. Paul Hospital Millennium Medical college can be attributed to a combination of factors. In addition to the hospital's geographical proximity to patients residing in the Oromia region, its role as a referral center for oncology care across a wide catchment area, including Oromia, underscores its capacity to provide specialized cancer services. This suggests that patients may choose St. Paul Hospital not only for its proximity, but also as it is one of the few tertiary government hospitals providing advanced pediatric oncology services with specialized expertise in Addis Ababa.

The age distribution of patients with solid tumors shows a significant concentration among those aged 1–5 years; other studies have reported higher prevalence in the 5–10 years age group [10, 11, 12, 13]. This age distribution reflects both the vulnerability of young children to malignancies and the challenges in diagnosing and managing cancer in this age group. Early detection strategies and community‐based awareness programs aimed at promoting early presentation to healthcare facilities could potentially improve outcomes by facilitating timely diagnosis and intervention.

### 4.2 Tumor Distribution and Characteristics

The tumor distribution observed in this study mirrors established patterns in pediatric oncology, with retinoblastoma, Wilms tumor, and rhabdomyosarcoma emerging as the most prevalent malignancies [9]. Patients with retinoblastoma account for 1.4% of children with cancer cared for at Gondar University, a tertiary care hospital in Northwestern Ethiopia with a pediatric hemato‐oncology service. At SPHMMC, we found a higher number of patients with retinoblastoma who comprised nearly one‐third of solid tumor diagnoses (30.6%). In part, this may be the result of our cancer center's ongoing commitment with a dedicated team of pediatric oncology and ophthalmology departments [7]. The high incidence of retinoblastoma underscores the importance of ophthalmologic screening programs for early detection and intervention [14]. Similarly, the predominance of Wilms tumor, often presenting with an abdominal mass, highlights the need for vigilant clinical evaluation and imaging modalities for prompt diagnosis.

The observed variations in tumor characteristics, such as staging and histological subtypes, have implications for treatment planning and prognostication. For instance, the predominance of advanced‐stage disease in certain tumor types underscores the importance of strategies to improve early detection and referral pathways, as demonstrated by our finding that most retinoblastomas were diagnosed in Reese–Ellsworth Group E, followed by Group C, as opposed to a study in Kashmir, India, where most tumors were diagnosed in Group B, followed by Group D. Similarly, most Wilms tumors in our sample were diagnosed in Stage IV, followed by Stage II, whereas the same study from Kashmir described most of the Stage III disease, followed by Stage I. In the case of rhabdomyosarcoma, we found that most patients were diagnosed in Stage III, followed by Stage IV. Numerous factors may account for the advanced stage of patients with RMS, including lack of awareness of clinical presentation among primary care providers and poor health‐seeking behavior in the community. Furthermore, the distribution of tumors across different anatomical sites underscores the heterogeneity of pediatric oncology and the need for tailored therapeutic approaches based on tumor location and biology.

### 4.3 Short‐Term Treatment Outcomes

The short‐term treatment outcomes reported in this study reflect the complex interplay of various factors, including tumor biology, treatment modalities, and patient‐specific factors. Although a notable proportion of patients achieved remission (14.7%), indicating successful treatment responses, the majority (55.1%) are still undergoing treatment, underscoring the prolonged nature of therapeutic interventions for pediatric solid tumors. Although only a small number of patients in our study experienced disease relapse (2.56%) this nonetheless highlights the need for vigilant monitoring and follow‐up care to promptly detect and manage disease recurrence. Additionally, the low rates of defaulters (1.28%) and loss to follow‐up (3.2%) suggest relatively good adherence to treatment regimens and follow‐up appointments. This is particularly notable when compared to reports from other pediatric oncology centers in East & South African regions. For instance, a study conducted at Moi Teaching and Referral Hospital in Kenya reported that 54% of children have abandoned treatment [15]. Additionally, a study assessing treatment outcomes and abandonment among a cohort of children diagnosed with cancer at the University Teaching Hospital (UTH) in Zambia found that only 8% completed their treatment regimen, with most patients either dying during treatment or abandoning care [16]. Nevertheless, the mortality rate of our study (13.4%) emphasizes the severity and potential lethality of pediatric solid tumors, necessitating ongoing efforts to improve treatment efficacy and outcomes. Strategies to address treatment‐related barriers, such as financial constraints, transportation issues, and psychosocial support needs, are imperative to optimize treatment adherence and overall outcomes.

Furthermore, the lack of radiotherapy services at our hospital has posed a significant hurdle in providing comprehensive cancer care. This deficiency may contribute to the rates of mortality and some patients opting to leave against medical advice and defaulting out of treatment.

## 5 Conclusion

This pioneering study evaluating pediatric solid tumors at a hemato‐oncology unit in a resource‐constrained LMIC reveals a diverse patient demographic, with retinoblastoma, Wilms tumor, and rhabdomyosarcoma being the most prevalent tumor types. Chemotherapy was the primary treatment modality, yielding notable remission rates, although some patients relapsed or defaulted on treatment. To build on these findings and optimize early detection, we advocate for a multi‐faceted approach. Specifically, the need to develop and implement comprehensive educational programs for healthcare professionals, conduct public awareness campaigns, and establish standardized guidelines and protocols within healthcare facilities. Furthermore, investing in infrastructure and introducing radiation therapy services can significantly enhance outcomes for patients with treatable solid tumors. By adopting these strategies, we can improve early detection, treatment, and survival rates for pediatric solid tumor patients.

## Author Contributions

D.A. participated in the conception and design of the study, data collection and analysis, interpretation of the findings, and authored the initial draft. M.D. participated in the conception and design of the study, analysis and interpretation of the findings, and drafting the manuscript. T.H., A.T.S., and Y.U. critically reviewed early drafts and participated in manuscript revisions. All authors read and approved the final manuscript.

## Ethics Statement

Ethical approval was granted by the SPHMMC Institutional Review Board. Informed written consent was not required, as the data were collected from secondary sources (medical records). Additionally, as the study relied on retrospective patient chart data, obtaining individual consent was not feasible.

## Conflicts of Interest

The authors declare no conflicts of interest.

## Data Availability

The datasets generated and/or analyzed during the current study are available from the corresponding author upon reasonable request.

## References

[1] E. Ward , C. DeSantis , A. Robbins , B. Kohler , and A. Jemal , “Childhood and Adolescent Cancer Statistics, 2014,” CA: A Cancer Journal for Clinicians 64, no. 2 (2014): 83–103. 24488779 10.3322/caac.21219

[2] L. A. G. Ries , M. A. Smith , J. G. Gurney , et al., eds., Cancer Incidence and Survival Among Children and Adolescents: United States SEER Program 1975–1995 (National Cancer Institute, SEER Program, 1999).

[3] E. Ward , C. DeSantis , A. Robbins , B. Kohler , and A. Jemal , “Childhood and Adolescent Cancer Statistics, 2023,” CA: A Cancer Journal for Clinicians 73, no. 4 (2023): 209–232, 10.3322/caac.21788. 24488779

[4] E. Steliarova‐Foucher , M. Colombet , L. A. G. Ries , et al., “International Incidence of Childhood Cancer, 2001–2010: A Population‐Based Registry Study,” Lancet Oncology 23, no. 4 (2022): 500–509, 10.1016/S1470-2045(21)00696-5. PMC5461370 28410997

[5] I. Qaddoumi , A. Musharbash , M. Elayyan , et al., “Closing the Survival Gap: Implementation of Medulloblastoma Protocols in a Low‐Income Country Through a Twinning Program,” International Journal of Cancer 122, no. 6 (2008): 1203–1206. 17985345 10.1002/ijc.23160

[6] N. Sharma , A. Ahmad , G. M. Bhat , S. A. Aziz , M. M. Lone , and N. A. Bhat , “A Profile of Pediatric Solid Tumors: A Single Institution Experience in Kashmir,” Indian Journal of Medical and Paediatric Oncology 38, no. 4 (2017): 471–477. 29333015 10.4103/ijmpo.ijmpo_95_16 PMC5759067

[7] S. Yifru and D. Muluye , “Childhood Cancer in Gondar University Hospital, Northwest Ethiopia,” BMC Research Notes 8 (2015): 474. 26404043 10.1186/s13104-015-1440-1 PMC4582631

[8] K. Rathi , A. Abhishek , K. Singh , and A. Bahadur , “Epidemiology of Paediatric Tumours at Tertiary Care Centre,” Indian Journal of Medical and Paediatric Oncology 28 (2007): 33–35, 10.1055/s-0041-1733214.

[9] Y. Tadesse and G. Etsegenet , “Pattern of Childhood Malignancies in a University Referral Hospital in Addis Ababa,” Ethiopian Journal of Pediatrics and Child Health 4 (2008): 27–31.

[10] T. Teka , “Childhood Malignancies in an Ethiopian Teaching Hospital,” Ethiopian Medical Journal 30, no. 3 (1992): 159–162. 1396618

[11] B. Chawla , F. Hasan , R. Azad , et al., “Clinical Presentation and Survival of Retinoblastoma in Indian Children,” British Journal of Ophthalmology 100 (2016): 172–178. 26061162 10.1136/bjophthalmol-2015-306672

[12] A. Singh , C. Shields , and J. Shields , “Prognostic Factors in Retinoblastoma,” Journal of Pediatric Ophthalmology and Strabismus 37 (2000): 134. 10845413 10.3928/0191-3913-20000501-04

[13] A. T. Atanda , L. J. Anyanwu , O. J. Atanda , A. M. Mohammad , L. B. Abdullahi , and A. U. Farinyaro , “Wilms Tumour: Determinants of Prognosis in an African Setting,” African Journal of Paediatric Surgery 12, no. 3 (2015): 171–176, 10.4103/0189-6725.170185. 26612121 PMC4955426

[14] K. M. Waddell , K. Kagame , A. Ndamira , et al., “Clinical Features and Survival Among Children With Retinoblastoma in Uganda,” British Journal of Ophthalmology 99, no. 3 (2015): 387–390, 10.1136/bjophthalmol-2014-305564. 25217695

[15] F. Njuguna , S. Mostert , A. Slot , et al., “Abandonment of Childhood Cancer Treatment in Western Kenya,” Archives of Disease in Childhood 99, no. 7 (2014): 609–614, 10.1136/archdischild-2013-305052. 24681695

[16] J. S. Slone , C. Chunda‐Liyoka , M. Perez , et al., “Pediatric Malignancies, Treatment Outcomes and Abandonment of Pediatric Cancer Treatment in Zambia,” PLoS One 9, no. 2 (2014): e89102. 24586527 10.1371/journal.pone.0089102 PMC3931678

